# Applications of artificial intelligence (AI) in diagnostic radiology: a technography study

**DOI:** 10.1007/s00330-020-07230-9

**Published:** 2020-09-18

**Authors:** Mohammad Hosein Rezazade Mehrizi, Peter van Ooijen, Milou Homan

**Affiliations:** 1 School of Business and Economics, KIN Center for Digital Innovation, Vrije Universiteit Amsterdam, De Boelelaan 1105, VU Main Building A-wing, 5th floor, 1081 HV Amsterdam, The Netherlands; 2 Department of Radiation Oncology, Coordinator Machine Learning Lab, Data Science Center in Health (DASH), University of Groningen, University Medical Center Groningen, Hanzeplein 1, 9713 GZ Groningen, The Netherlands

**Keywords:** Artificial intelligence, Radiology, Workflow, Radiologists, Forecasting

## Abstract

**Objectives:**

Why is there a major gap between the promises of AI and its applications in the domain of diagnostic radiology? To answer this question, we systematically review and critically analyze the AI applications in the radiology domain.

**Methods:**

We systematically analyzed these applications based on their focal modality and anatomic region as well as their stage of development, technical infrastructure, and approval.

**Results:**

We identified 269 AI applications in the diagnostic radiology domain, offered by 99 companies. We show that AI applications are primarily narrow in terms of tasks, modality, and anatomic region. A majority of the available AI functionalities focus on supporting the “perception” and “reasoning” in the radiology workflow.

**Conclusions:**

Thereby, we contribute by (1) offering a systematic framework for analyzing and mapping the technological developments in the diagnostic radiology domain, (2) providing empirical evidence regarding the landscape of AI applications, and (3) offering insights into the current state of AI applications. Accordingly, we discuss the potential impacts of AI applications on the radiology work and we highlight future possibilities for developing these applications.

**Key Points:**

• *Many AI applications are introduced to the radiology domain and their number and diversity grow very fast.*

• *Most of the AI applications are narrow in terms of modality, body part, and pathology.*

• *A lot of applications focus on supporting “perception” and “reasoning” tasks.*

**Electronic supplementary material:**

The online version of this article (10.1007/s00330-020-07230-9) contains supplementary material, which is available to authorized users.

## Introduction

### The need for systematically analyzing the AI developments

Artificial intelligence (AI) is defined as “an artificial entity ... able to perceive its environment .... search and perform pattern recognition ... plan and execute an appropriate course of action and perform inductive reasoning” (p. 246) [1]. For the last few years, there have been many discussions in the radiology community regarding the potentials of AI for supporting medical diagnosis and numerous research projects have used AI for answering medical questions [1–3].

There have also been many AI applications offered to the market, claiming that they can support radiologists in their work [4]. However, the clinical applications of AI in daily practice are limited [3]. Why is there a major gap between the promises of AI and its actual applications in the domain of radiology?

Part of the answer lies in the long way that these applications need to go through before they can be effectively used in the clinical settings. It is important to systematically review these applications, scrutinize their functionalities, their state of development and approval, and how they can be integrated into the radiology workflow.

Various opinion papers [1, 2, 8] and white papers [9] have suggested many potential use cases of AI for radiology. Yet, we lack a systematic, comprehensive overview of the extent these possibilities have already been developed into applications and how far these applications are validated and approved? Hence, we need to critically and systematically examine where the current AI applications mainly focus on and which areas of radiology work are still not touched, but are going to be addressed.

Such an analysis should be conducted by scientific communities, to be based on systematic methods, and hence be replicable and transparent to the public discussions. In addition, we need to critically reflect on the technological applications, without having interests in promoting certain applications. This way we can engage radiologists in thinking about the relevant use cases and shaping future technological developments.

This systematic review, so-called *technography*,^1^ is essential for two reasons. First, despite the wide range of studies that discuss the various possibilities of AI [1, 2], we do not know to what extent and in which forms these possibilities have been actually materialized into applications.

Second, AI applications in the radiology domain are in an “emerging” phase. Many functionalities and use cases are yet to be developed, critically evaluated in practice, and complemented by the subsequent developments [7]. Therefore, the researchers, developers, and medical practitioners need to trace and critically evaluate the technological developments, detect potential biases in the way these applications are developed, and identify further opportunities of AI applications.

In the next sections, we lay out the framework based on which we examine the AI applications in the domain of diagnostic radiology. Then, we report our technography study. Finally, we discuss the implications of our findings.

## Methods

Similar to a systematic literature review, we conducted a systematic review of AI applications in the domain of radiology. We started by searching for all relevant applications presented during RSNA 2017 and RSNA 2018, ECR 2018, ECR 2019, SIIM 2018, and SIIM 2019. We also consulted market survey reports (e.g., [12]), technical blog posts, news, and published articles. An application was selected when it has been developed for supporting activities in the diagnostic radiology workflow *and* claims to have learning algorithms such as convolutional neural networks. The data is up to date as of August 2019.

To focus on the diagnostic radiology, we excluded the applications that merely offer a marketplace for other applications, or merely act as a connection between RIS and PACS, or do not work with any medical imaging data. We also excluded the applications that do not explicitly refer to any learning algorithm (e.g., when it is generally said it is “advanced analytics”). We also excluded or corrected for cases that were discontinued or merged. This process eventually resulted in 269 applications, offered by 99 companies (see Appendixes 1 and 2 for the full list of included and excluded applications).

For each application, we collected a rich set of data about its (1) developing company, (2) features and functionalities, (3) ways of being implemented and used, and (4) legal approval. We collected this data from multiple sources such as company websites, press releases, FDA approval documents, white papers, YouTube videos, user manuals and guidelines, and scientific articles. We also cross-checked different sources and checked the credibility of the issuing sources (e.g., formal regulatory agencies such as FDA).

### Data analysis

We build on four questions in our analysis of AI applications.

#### Who are the active developers of AI applications?

At the macro-level, it is important to know the popularity and diversity of the AI applications and the companies that are active in offering them. This overview shows us the overall trends in the development of AI applications across different regions.

#### What are the states of development and legal approval of AI applications?

AI applications can be in different development stages such as “under development,” “under test,” and “approved.” Mapping the applications across these stages shows the progress of the AI developments. Moreover, AI applications are often subject to Medical Device Regulations (MDR). They have to be approved by regulatory authorities before they can be clinically used. It is interesting to see how extensively and strictly these applications are approved.

#### How narrow are the AI applications?

Many AI applications are designed to address a very specific task, work with images taken from a particular modality (e.g., only on the MRI scans), examine a particular anatomic region (e.g., brain or lung), and answer a specific medical question (e.g., detecting lung nodule) [7, 8]. This narrowness has been a concern regarding the practicality and value of these applications [8]. We examine the extent to which the AI applications are narrow in terms of their focal modality, anatomic region, and medical task. We also examine how these applications are offered to the users (e.g., as cloud-based or on-premise) and integrated into the radiology workflow.

#### What types of radiology tasks do AI applications focus on?

AI applications are often claimed to be good at supporting tasks that are quantifiable, objective, and routine [10]. The tasks these applications target have a major consequence on their impacts on the radiology work [11]. In the case of radiology, this can be reflected in the focus of AI applications on the various tasks in the workflow process, namely acquisition, processing, perception, reasoning, and reporting, as well as administration (e.g., scheduling, referral, notification of the follow-up). However, the functionalities that developers may see feasible are not necessarily the ones that radiologists may find effective for their work. It is important to examine which areas of radiology workflow are mainly targeted by the current AI applications and what are the untapped opportunities for future developments.

In this process, we first developed the codebook that guided our coding and ensured the consistency of coding across the research. We followed the procedure of deductive “content analysis” [13] to code for a range of dimensions (see Table 1).

**Table 1:** The codebook for analyzing the AI applications

Concept	Items
Functionalities	The features and values that solutions offer that could impact diagnostic accuracy
Focal modality	CT, MRI, X-ray, echocardiography, mammography, ultrasound
Targeted step in the workflow	Administration, acquisition, processing, perception, reasoning, reporting
Focal anatomic region	Brain, breast, lung, cardio, liver, spine, skeletal, thyroid, prostate, chest, abdomen, fetal
Developmental stage	Development: when the algorithm is being designed and developed and has been trained, but is not yet validated Testing: when the algorithm is under the validation tests in various real data bases Marketed: when the application is approved and therefore has been formally introduced to the market
Regulatory approval	FDA approval, FDA clearance, CE marked, etc.
Introduction date	The year that the application is offered to the market
Company foundation	When the company is established
Company location	The country and region of the company

We conducted our analysis by examining various patterns across the applications based on the abovementioned dimensions through cross-tabulation [14]. The quantified patterns were then interpreted based on qualitative data.

## Findings

### Who are active developers of AI applications?

**Fig. 1:**
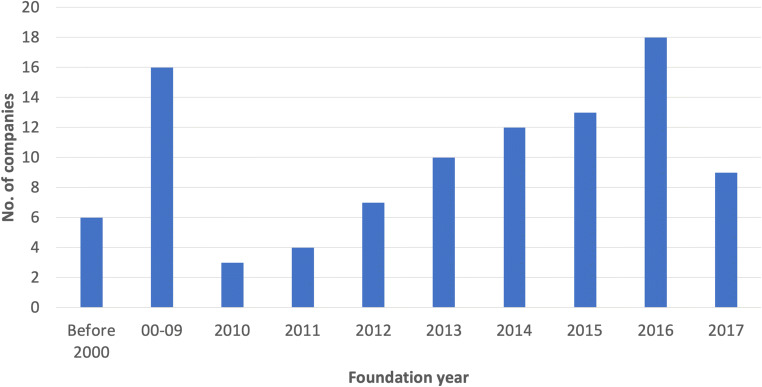
The foundation date of companies active in the market

We identified 269 applications as of August 2019. Compared with 146 applications in December 2018, this number doubled in half a year. These applications are offered by 99 companies, from which 75% are founded after 2010 (Fig. 1). Startups are increasingly dominant in this market.

**Fig. 2:**
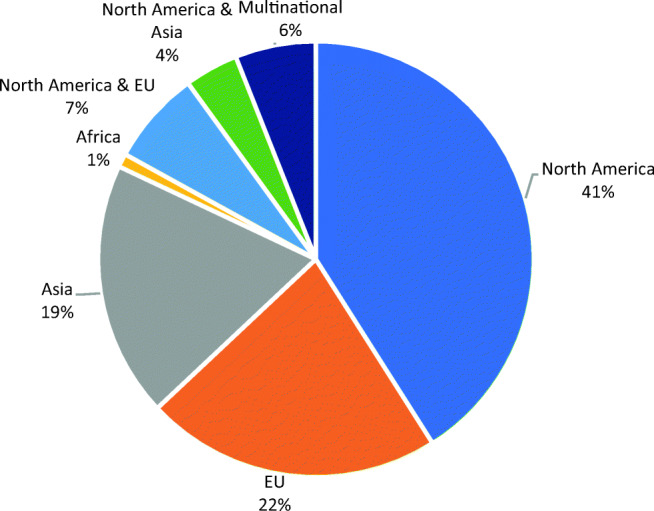
The share of applications developed in various geographical markets

As shown in Fig. 2, North America (NA) is the most active market. Next to European companies, Asian companies are also active in this market.

### What are the states of development and legal approval of AI applications?

In our sample, 56% of the applications are commercially available in the market, while 38% are in the “test” and 6% in the “development” phases. For the known cases (67%), 32% are offered as “only cloud-based” and 4% as “only on-premise,” but 46% are offered as both cloud-based and on-premise. A total of 54% of the applications are accessible via PACS/RIS, whereas 25% are offered as stand-alone applications.

More than half of the applications (60%) do not have any regulatory approval,^2^ from which 60% are cleared by FDA, 62% are CE-marked, and 32% by both FDA and CE. The trend of receiving regulatory approval shows a sharp increase in the last 2 years. Some countries such as Korea and Canada have their own regulatory authorities.

### How narrow are the AI applications?

**Fig. 3:**
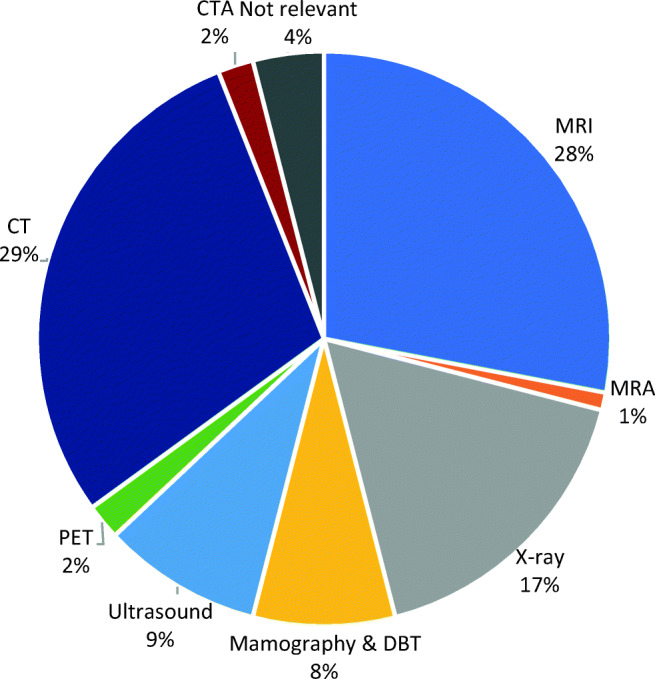
The relative share of applications based on their targeted modalities

Most of the AI applications target “CT,” “MRI,” and “X-ray” modalities. Very few applications work with “ultrasound” (9%) and “mammography” (8%) modalities (Fig. 3). Most of the applications (95%) work with only one single modality. Only eight applications (3%) work with both CT and MRI modalities.

### The focal anatomical regions

**Fig. 4:**
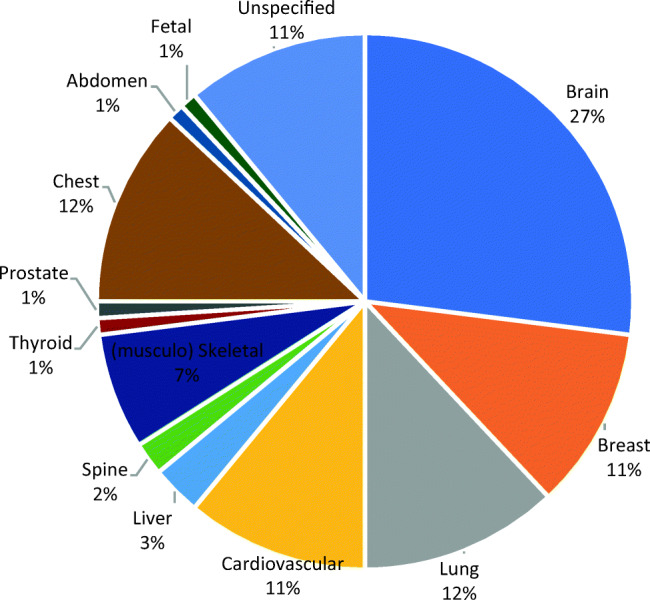
The share of applications focusing on a specific anatomic region

The applications very often (95%) target one specific anatomical region. Only four applications work with the images taken from multiple anatomic regions or organs (e.g., both lung and heart).^3^ As Fig. 4 shows, the “brain” is the most popular organ. This seems to be partly due to the prevalence of MRI scans and the very large cohort of algorithms that examine neurological diseases such as Alzheimer.

The anatomic regions related to the “Big-3” diseases (lung cancer, COPD, and cardiovascular diseases) are the next most popular organs that these applications target, which are often examined via CT scans. Due to the prevalence of the data from breast cancer screening, the breast is a popular anatomic region. The liver, spine, thyroid, and prostate are far less frequently targeted by these applications. Applications that target the liver, spine, skeletal, and thyroid are primarily in the development and test stages. Finally, very few applications focus on fetal images, which seems to be due to the challenges of analyzing ultrasound inputs and because the digitalization of ultrasound modality is lagging behind.

### What types of radiology tasks do AI applications focus on?

**Fig. 5:**
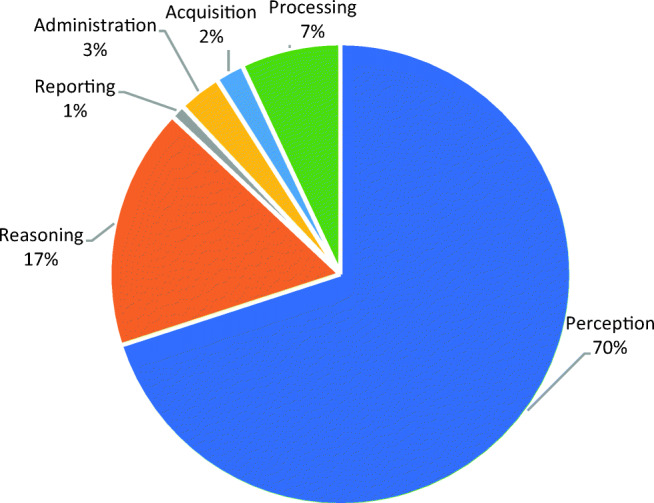
The relative share of applications based on their targeted workflow tasks

The AI applications primarily target “perception” and “reasoning” tasks in the workflow. Only a few applications address “administration” and “reporting” tasks (Fig. 5). In the following paragraphs, we dig into the functionalities that applications offer for supporting radiology tasks.

#### Administration

A few applications support the referring doctors and radiologists for deciding on the relevant imaging examinations (e.g., which modality or radiation dosage) by analyzing patients’ symptoms and the examinations that were effective for similar patients. This way, radiologists can avoid unnecessary examinations and perform evidence-based examinations. A few applications also support the scheduling and balancing the workload of radiologists.

#### Acquisition

Some applications monitor the uptime and performance of machines and offer (predictive) insights into e.g. the expected maintenance time. Some other applications assess the quality of the acquired images to ensure that the target organs are properly covered, their boundaries are clear, and they do not miss important informational elements. This way, these applications enhance the efficiency and pace of the acquisition process.

#### Processing

Several applications support the processing of the images to improve their quality (e.g., on clarity, brightness, and resolution) in the post-acquisition stage. These applications enable technicians with lower skills to still produce good-quality images, reduce the need for repeating the acquisition, and lower the radiation without compromising the image quality. In addition, they facilitate the comprehension of the images by the doctors in the subsequent stages.

#### Perception and reasoning

A majority of the applications offer functionalities that support the *perception* and *reasoning tasks*. As presented in Table 2, we can categorize these functionalities into seven categories.

**Table 2:** AI functionalities related to the perception and reasoning

AI functionality	The supporting role in the radiology workflow
Segmentation (8%*)	Segmentation is a functionality of many applications that designate a specific organ. The segmentation not only liberates the radiologists from this task, but also optimizes the limited attentional resources that radiologists have during the work and potentially reduces both false-positive and false-negative errors by supporting them to focus on the most relevant part and the image. This is sometimes achieved through suppressing less relevant aspects of the image to reduce the information overload and thus focuses the attention of radiologists on the more important aspects of the image.
Quantification and extraction of features (28%)	Many of the applications quantify certain aspects of the image (e.g., bone density), measure some aspects of the organ (e.g., brain volume), or extract quantitative features from the image (e.g., the level of coronary calcium scores). The outcome is often presented as numbers and charts (when it is done on a series of images).
Detecting and highlighting the suspicious areas (42%)	This category of functionalities focuses on a particular pathology or abnormality and looks for their signs (e.g., nodules, strokes, and high-density tissues) and highlights them. These applications are often trained for a particular (common) disease and aim to ensure the accurate examination of the images and to help radiologists detect and decide about certain problems.
Comparison, cross-referencing, and longitudinal analysis (8%)	As one step further to provide medical insights, some applications compare the different images of one patient to detect the changes in certain aspects over time (e.g., tumor size). This function is sometimes used to find other similar cases that have been previously diagnosed and therefore providing insights into further consultations and comparisons by the radiologist.
Diagnosis and classifying abnormalities (11%)	Diagnosis is a common functionality that builds on the previous functionalities, but combine them with a judgment regarding the likelihood of certain problems. This judgment can be achieved by comparing with the normal/healthy standards (e.g., the normal brain size), as well as identifying certain problematic areas (e.g., broken bones or tumors). These applications vary depending on how much they frame their outputs as “the actual diagnosis” or as “pre-diagnosis” to be further examined by the doctors.
Prognosis (2%)	Only a few applications offer the possibility of predicting the likelihood of certain diseases or problems based on the inspection of the current examinations. This prognosis is often focused on a particular problem and sometimes uses additional clinical information, next to the information that is extracted from the image.
Patients profiling and synopsis, and case prioritization (1%)	A group of applications actually do not work directly with the images, rather extract additional information related to the patient from the previous reports and electronic medical records, next to each patient’s image (e.g., as dashboard or personalized view about a patient). The analytical insights that they offer enable radiologists to have a broader overview about the patient’s history and conditions and therefore more accurately examine their images.

*Percentages reflect the share of applications having this functionality

#### Reporting

Although several applications produce their outputs in the forms of free text, tables, and graphs, some applications are dedicated to reporting. They assist in producing more accurate and faster transcription, generating structured reports, reminding radiologists on the list of critical aspects to be checked, and signaling the probable differential diagnoses.

## Discussions

Our study offers an objective overview of the AI applications in the diagnostic radiology domain, their stages of development and legal approval, and their focus regarding imaging modalities, pathologies, and clinical tasks. These applications offer many functionalities, yet each focus on a very specific modality, narrow medical question, and a specific anatomic region. This picture objectively demonstrates the fact that current AI applications are still far from being comprehensive.

The fact that mainly startups are active in the market shows that still a lot of the applications are based on the entrepreneurial exploration, originated from technology-driven ideas, and often driven by the availability of data and technically feasible use cases. Similar to other similar markets, larger (medical) companies may gradually become more active and enhance the scale of the investments and technological resources.

Still, a large portion of the AI applications are yet to be approved. Even the ones that are approved often do not have a strict approval (e.g., only one application has FDA “approval” and the rest have FDA “clearance”) and they get the approval for limited use cases (e.g., as tentative diagnosis without clinical status). Given the new legislations such as Medical Device Regulations, AI applications are expected to undergo stricter approvals.

AI applications are quite narrow in terms of the modalities, anatomic regions, and tasks. This narrowness of AI applications can limit their applicability in the clinical practice. We see some companies try to partner with other companies to offer a wider range of applications. There are some platforms that try to integrate various AI applications. However, still the users need to choose from a long list of applications, each with a narrow functionality. Further integration of the existing applications into the regular workflow of radiologists (e.g., running in the background of the PAC systems) may enhance the effectiveness of the AI applications.

Future developments may focus on applications that can work with multiple modalities and examine multiple medical questions. Should the developers prioritize multi-modality over multi-pathology? For instance, does the market prefer an algorithm that is capable of working with both MRI and CT scan images, but only for detecting tumors (multi-modal single-pathological solution), over an algorithm that is capable of checking various problems such as nodules, calcification, and cardiovascular disorders, all in one single chest CT (single-modal multi-pathological solution)? Perhaps the answer depends on the implementation context (e.g., clinical examination vs. population study) and the way the clinical cases are allocated (e.g., based on the modality or diseases). Our observation suggests that still this is an open question for many developers and we do not see a visible trend in the market.

Our analysis also shows that the algorithms that are in the market limitedly use the “clinical” and “genetic” data of the patients. There are ample opportunities for applications that integrate other sources of data with the image data to enrich, validate, and specify the insights that can be derived from the images. For that, standardization of the data exchange and interoperability of medical systems are two key challenges.

Only a handful of the current applications offer “prognosis” insights. In the future, AI applications may deploy predictive analytics to support preventive healthcare services. We see that the main focus of AI applications is on diagnosing various pathologies. Yet, only a small portion of the applications target “administration” tasks such as scheduling, prioritizing, and reporting, which can be very effective for supporting radiologists in their work and often do not require strict clinical approvals.

Finally, when these applications have a narrow scope, the effort and time that radiologists need to spend on launching and using these applications may outweigh their benefits. Therefore, it is important that AI applications are seamlessly integrated in the daily workflow of the radiologists. Our analysis shows that AI applications often do not afford “bi-directional interactions” with the radiologists for receiving real-time feedback. Similar to other successful learning algorithms (e.g., navigation tools), the feedback process needs to be implemented as a natural part of using these systems. The current legal approval paradigm is a challenge since it demands “fixation” of the algorithms, which can hinder improvement of the AI applications during their actual use. New legal initiatives need to embrace constant performance tracking and continuous improvements of the applications.

## Electronic supplementary material

ESM 1 (DOCX 16 kb)

ESM 2 (XLSX 284 kb)

## Abbreviations

AI: Artificial intelligence
CE mark: European Conformity Marking
COPD: Chronic obstructive pulmonary disease
CT: Computed tomography
ECR: European Conference of Radiology
EU: European Union
FDA: Food and Drug Administration
MRI: Magnetic resonance imaging
NA: North America
PACS: Picture Archiving and Communication System
RIS: Radiological Information System
RSNA: Radiological Society of North America
SIIM: Society for Imaging Informatics in Medicine
US: United States
X-Ray: X-Radiation

## Footnotes

1 Technography, also called the study of technological developments in a domain of application, is a well-established approach to systematically analyze the technological trends, the dominant approaches in designing technologies, and the ways in which technology is getting shape over time. For some examples of these studies, see, e.g., [5, 6].

2 For around 5% of the applications that are related to the administration of the workflow, medical approval is not needed.

3 For some applications that focus on the administration, reporting, and image enhancement, the focus on the anatomic region is not relevant.
