# Supplementary material for: Applications of artificial intelligence (AI) in diagnostic radiology: a technography study
Source: Eur Radiol. 2020 Sep 18;31(4):1805–11. doi: 10.1007/s00330-020-07230-9 (PMC7979626; doi:10.1007/s00330-020-07230-9)
Supplement: Supplementary file 1 — (DOCX 16 kb) [file 330_2020_7230_MOESM1_ESM.docx]

Table A1. Summary of the excluded applications and the reasons

| **Exclusion reason** | **Count** |
| --- | --- |
| Marketplace for other applications | 10 |
| Connection between RIS and PACS | 4 |
| Do not work with any medical imaging data | 12 |
| No clear reference to AI in their description | 26 |
| Discontinued, merged, or bought by other companies | 15 |
| Focused on a different area of radiology | 16 |
| Not enough information | 11 |
| Teleradiology (offer the service) | 5 |
| SDKs and OEMs | 11 |
| Extending their own hardware | 2 |
| **Total** | **112** |
